# Risk Factors for Hospital and Long-Term Mortality of Critically Ill Elderly Patients Admitted to an Intensive Care Unit

**DOI:** 10.1155/2014/960575

**Published:** 2014-12-16

**Authors:** A. Mukhopadhyay, B. C. Tai, K. C. See, W. Y. Ng, T. K. Lim, S. Onsiong, S. Ee, M. J. Chua, P. R. Lee, M. L. Loh, J Phua

**Affiliations:** ^1^Division of Respiratory and Critical Care Medicine, University Medicine Cluster, National University Health System, Singapore; ^2^Department of Medicine, Yong Loo Lin School of Medicine, National University of Singapore, Singapore; ^3^Saw Swee Hock School of Public Health, National University of Singapore and National University Health System, Singapore; ^4^The University of Sheffield, Sheffield, UK; ^5^National University of Singapore, Singapore

## Abstract

*Background*. Data on long-term outcomes of elderly (≥65 years) patients in ICU are sparse. *Materials and Methods*. Adult patients (*n* = 1563, 45.4% elderly) admitted over 28 months were analyzed by competing risks regression model to determine independent factors related to in-hospital and long-term mortality. *Results*. 414 (26.5%) and 337 (21.6%) patients died in-hospital and during the 52 months following discharge, respectively; the elderly group had higher mortality during both periods. After discharge, elderly patients had 2.3 times higher mortality compared to the general population of the same age-group. In-hospital mortality was independently associated with mechanical ventilation (subdistribution hazard ratio (SHR) 2.74), vasopressors (SHR 2.56), neurological disease (SHR 1.77), and Mortality Prediction Model II score (SHR 1.01) regardless of age and with malignancy (SHR, hematological 3.65, nonhematological 3.4) and prior renal replacement therapy (RRT, SHR 2.21) only in the elderly. Long-term mortality was associated with low hemoglobin concentration (SHR 0.94), airway disease (SHR 2.23), and malignancy (SHR hematological 1.11, nonhematological 2.31) regardless of age and with comorbidities especially among the nonelderly. *Conclusions*. Following discharge, elderly ICU patients have higher mortality compared to the nonelderly and general population. In the elderly group, prior RRT and malignancy contribute additionally to in-hospital mortality risk. In the long-term, comorbidities (age-related), anemia, airway disease, and malignancy were significantly associated with mortality.

## 1. Introduction

A natural corollary of the universal increase in life expectancy is a rapidly aging population [1, 2]. Worldwide there are now 70 million people aged 80 years or above, and the number is expected to increase five-fold by 2050. Today, the older population grows faster than the general population and by 2025–2030, the population of elderly over 60 years old will grow 3.5 times as rapidly as the total population [1]. In Singapore, the percentage of elderly (≥65 years) increased from 6% in 1990 to 9.9% in 2012 [3].

Greater healthcare utilization by the growing elderly population is likely to put a strain on the hospital services including the intensive care units (ICUs). Eleven percent of Medicare recipients in the United States spend more than 7 days in the ICU within the last 6 months of life [4]. In 2004, 1 in 5 American patients died in the ICU, and the doubling of patients above the age of 65 years by 2030 is expected to significantly stretch ICU services [5]. Data from the Committee on Manpower for Pulmonary and Critical Care Societies (COMPACCS) suggest a progressive shortage of intensivist hours from 2007, reaching 35% by 2030, which is mainly driven by the growth of the elderly population [6].

Given the increased demand by the aging population in presence of resource limitations, it is important to know the outcomes of elderly patients admitted to the ICU and factors contributing to these outcomes. Outcomes of elderly populations have previously been studied [4, 7, 8], but aside from a large dataset of Medicare beneficiaries in the United States [9], most reports are restricted to small patient groups or preselected geriatric cohorts. Knowledge of long-term outcomes of elderly ICU patients is also limited as most studies have data for only 1-2 years following discharges from the hospital [10, 11]. With increased life expectancy, such longer-term data become highly relevant. With these considerations, we systematically studied a large prospective cohort of patients admitted to our medical ICU and high dependency unit (HDU). Our aims were to investigate their in-hospital and long-term (up to 52 months) mortality and to compare factors contributing to the mortality of elderly (≥65 years) versus nonelderly patients.

## 2. Materials and Methods

### 2.1. Setting

The National University Hospital is a 1000-bed tertiary academic medical center affiliated to the National University of Singapore. It has a 20-bed combined medical ICU/HDU that admits all patients under the medicine cluster, including hematology-oncology but excluding cardiology services. Patients are admitted from the emergency medicine department, acute hospital wards, and other units. Both the ICU and the HDU are covered by the same group of physicians and nursing staff. Physicians' (including 2 consultants (trained intensivists) and 2 fellows and 5-6 residents) cover is from 8 a.m. to 5 p.m. Out-of-hours services are covered by one fellow and two resident, with a consultant on-call off-site. The nurse-to-patient ratio varies from 1 : 1 (ICU) to 1 : 2 (HDU). From here on, use of the term ICU will refer to the combined ICU/HDU.

### 2.2. Study Design and Patients

In this prospective observational cohort study, all adult (≥18 years) patients who were admitted to ICU from January 2008 to April 2010 were included. Patients were divided into two groups: ≥65 (elderly) and <65 years of age (nonelderly) [11–13].

### 2.3. Data Collection

We used IntelliVue Clinical Information Portfolio (ICIP, Philips Healthcare), which records all critical care data prospectively in real time, as the study's ICU data source. We collected mechanical ventilation (MV), noninvasive ventilation (NIV), renal replacement treatment (RRT), and vasopressor usage from ICIP. The Mortality Prediction Model 0 (MPM) II scores on admission were calculated from the available data [14]. A hospital-wide computerized database (Computerized Patient Support System, CPSS, Singapore) that collects all the electronic records, including discharge summaries and biochemical, hematological, microbiological, and radiological investigations, was used to record the following data: demographics, comorbidities, preadmission conditions, admission source, length of stay, diagnosis, investigations (hematology and biochemistry), and outcomes (ICU and hospital). We collected mortality data for up to 52 months following the patients' discharge from the National Registry of Diseases Office, Singapore, where all deaths occurring in the country are recorded. To ensure data integrity and quality assurance, the data were checked extensively for accuracy and completeness, including outliers.

### 2.4. Statistical Analysis

Categorical baseline characteristics were compared using the Fisher's exact test. For admissions data where a subject may have multiple admissions, skewed continuous and categorical variables were evaluated using quantile and logistic regression accounting for individual patient as cluster. For analysis of mortality outcomes, survival time was calculated from the date of first admission to the date of death. Patients who were alive were censored on April 30, 2012, when the survival information was obtained from the National Registry of Diseases Office. In the analysis of in-hospital mortality, mortality after discharge from the hospital was considered as a competing risk, and vice versa. Cumulative incidence curves were compared between the two age groups using the competing risk methods [15]. The competing risks regression model was used to identify risk factors affecting mortality in the hospital and following hospital discharge [16]. The effects of these risk factors were quantified using the subdistribution hazard ratio (SHR) estimates and the associated 95% confidence interval (CI). Factors that were significant at the 5% level in the univariate analysis were further considered for inclusion in the multivariable competing risks regression model. Age-specific standardized mortality ratio (SMR) was calculated based on the mortality rate in the Singapore standard population and average follow-up duration [3]. All statistical analyses were generated using STATA version 12 (StataCorp LP, College Station, TX, USA) assuming a two-sided test at the conventional 5% level of significance.

The study was approved by the hospital's institutional review board (IRB) and ethics committee (Domain Specific Review Board, National Healthcare Group ref: 2011/01647) and requirement of consent was waived.

## 3. Results

**Figure 1:**
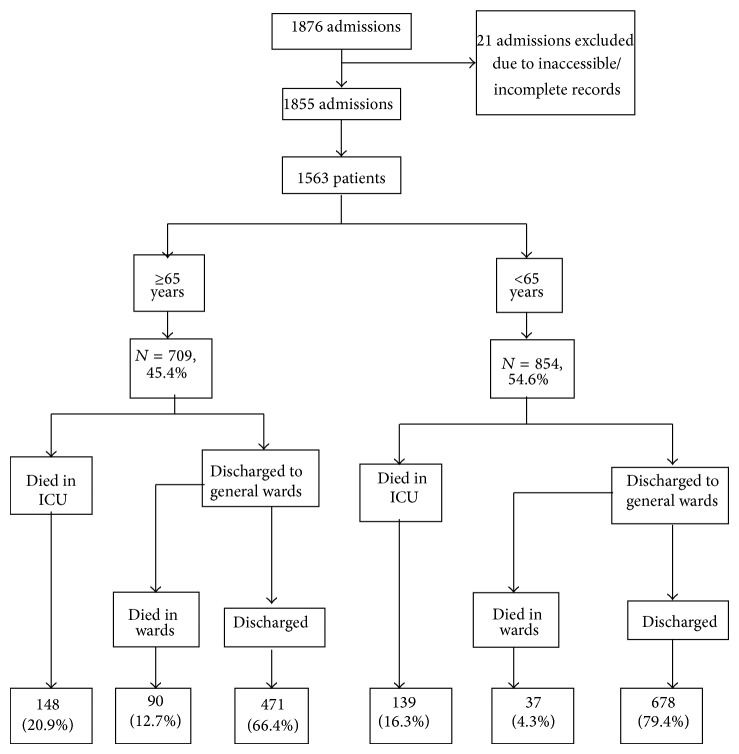
Patient inclusion.

In total, 1855 admissions in 1563 patients were available for analysis (Figure 1). Of these, 709 (45.4%) patients were ≥65 years of age. Baseline characteristics of the subjects according to age are shown in Table 1, while Table 2 summarized the information obtained at each admission. The median duration of follow-up was 3.1 (IQR 2.5–3.7) years.

**Figure 2:**
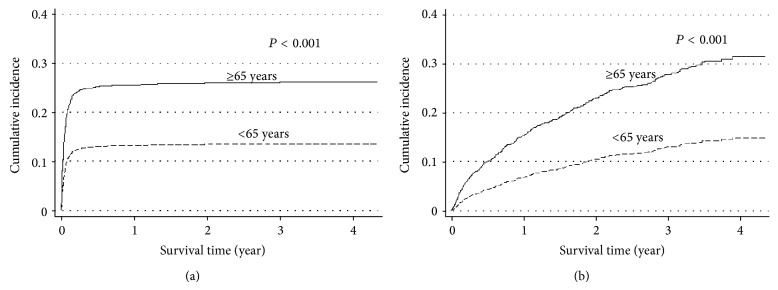
Cumulative incidence of mortality (a) in-hospital and (b) following hospital discharge according to age groups.

Four hundred and fourteen (26.5%) patients died during their hospitalization (including 287 (18.4%) ICU deaths) and 337 (21.6%) patients died following discharge from the hospital. Elderly patients had significantly higher mortality in both the ICU (20.9 versus 16.3%, *P* = 0.02) and hospital (33.6 versus 20.6%, *P* < 0.001).  Figure 2 shows the cumulative incidence of mortality in-hospital and after discharge from the hospital, both of which were markedly higher in the elderly group (all *P* < 0.001). In addition, the difference in mortality rates widened between the two groups over time following discharge from the hospital.

Table 3 describes the variables which were associated with in-hospital mortality and mortality following the patients' discharge from the hospital on univariate analyses.

Table 4 lists the independent predictors of in-hospital mortality on multivariate analysis. A neurological disease, use of MV and vasopressors, and higher MPM II score portend similar increases in in-hospital mortality for both the elderly and the nonelderly groups. Malignancy and RRT before admission predicted in-hospital mortality in only the elderly population. An endocrine diagnosis and use of NIV were protective in both groups, the latter conferring greater reduction in hazard in the elderly.

Table 5 details the independent predictors of mortality following discharge from the hospital on multivariate analysis. Airway disease, a nonhematological malignancy, and a low hemoglobin level were independently associated with a higher risk of death in both age groups. The same applies to the number of comorbidities, although this association was stronger for the nonelderly than the elderly group.

Table 6 shows the SMR between two groups after hospital discharge. Elderly patients who were admitted to ICU continued to have 2.3 times higher mortality compared to the general population in this age group.

## 4. Discussion

The important features of our study are that we included a large cohort of both elderly and nonelderly patients, provided long-term outcome data of more than 4 years, elucidated the independent predictors of in-hospital versus post-discharge mortality using a competing risks analysis and compared mortality of the elderly ICU patients with general population in the same age group following hospital discharges. Elderly patients (≥65 years) had higher mortality in the ICU and hospital than the nonelderly, but two-thirds of them survived until they were discharged from the hospital. In the long-term, elderly patients had much higher mortality, with the difference in mortality between the elderly and nonelderly widening over time. For in-hospital mortality, predictors which were common to both groups included neurologic disease, use of MV and vasopressors, and a higher MPM II score, while predictors which were restricted to the elderly group included malignancy and RRT. A different set of predictors were found for mortality following discharge from the hospital and this included low hemoglobin level on admission, airway disease, nonhematological malignancy, and the number of comorbidities. Importantly, following hospital discharge elderly patients have 2.3 times higher mortality in the long-term compared to similar age group in the general population.

Although the demographic definition of old age varies considerably, a general cutoff at 65 years is used in the vast majority of studies [3, 11–13]. Applying the same cutoff, we found that more than 45% of the admissions to our ICU were elderly. This proportion was consistent with those found in previous reports, such as the databases that generated the Acute Physiology and Chronic Health Evaluation (APACHE) II (24–54% in different centers) and the APACHE III (48%) scores [17, 18]. The case mix of the elderly patients differed from that of the nonelderly in our study, with more cases of airway (including chronic obstructive pulmonary disease (COPD)) and cardiovascular diseases, but less endocrine diseases (including diabetic ketoacidosis) (Table 1). This difference in case mix has an influence on the treatment modalities and outcome. Indeed, a significantly higher proportion of elderly patients received NIV for COPD; those who were admitted with neurological diseases had worse outcomes, and those with endocrine problems had better outcomes.

While we did not find any differences in the ICU and hospital lengths of stay between the two groups, mortality rates in the hospital and post-hospital discharge were higher in the elderly group than in the nonelderly group. In comparison to the short follow-up period of previous smaller studies of 3 months to 2 years [11, 19], our study included long-term outcome data of more than 4 years, with a median duration of over 3 years. The difference in mortality between the elderly and the nonelderly widened over time, and more than 30% of the elderly hospital survivors had died by the end of study period (Figure 2). This finding is further supported by the age-specific SMR which was calculated based on the mortality rate of the local general population for 2011 (Table 6). Elderly patients who were admitted to the ICU have 2.3 times higher mortality compared to the general population in the same age group. Nonelderly patients also have a higher post-hospital discharge SMR but this is likely related to the very low mortality in this age group in the general population. Similarly, Wunsch and colleagues found a 3-year mortality of 39.5% among a large cohort of elderly ICU survivors [9].

Previous studies have attempted to improve prognostication for elderly ICU patients, but it remains unclear if predictors of mortality differed between the elderly and the nonelderly [20]. In our study in-hospital mortality and long-term mortality were affected by different predictors. In-hospital mortality was largely determined by the severity of acute illness which is not surprising given similar findings in previous studies [21, 22]. We did not find any residual effect of these organ failures on long-term outcomes, which is in contrast to the findings of Lone and Walsh [23]. On the other hand, long-term mortality was predicted by the number of comorbidities, especially airway disease (COPD) and malignancy. In a previous retrospective analysis, we found a high long-term mortality rate despite a relatively good short-term outcome among COPD patients admitted to the ICU [24]. It is important to note that NIV, which was protective in the short term, might have preselected patients with COPD for long-term follow-up in our study. Interestingly, we also found for the first time that each 1 g/dL decrease in hemoglobin on ICU admission was associated with a 6% increase in the risk of mortality following discharge from the hospital. We postulate that this association may be accounted for by the fact that anemia is an indicator of poor general health and chronic illness.

It is important to consider how the current findings can be used by the clinicians, hospital administrators, researchers, and policymakers. Age is a well-known discriminatory factor during triage for admission to the ICU [2, 25] and elderly subjects often receive less aggressive treatment [26]. Many factors come into play in the decision to admit an elderly patient to ICU, including premorbid status, dependency in activities of daily life, availability of treatment modality, and importantly, the probability of a favorable outcome [22]. Our findings, that although two-thirds of elderly ICU patients survive till hospital discharge, one-third of these survivors will die in the next 3 to 4 years, especially if they have multiple comorbidities and advanced airway and malignant diseases, will thus serve to inform future patients, their families, and the healthcare professionals. Clinicians and hospital administrators should consider these risks when formulating local ICU admission policies, although whether such data will be perceived favorably or regarded as poor outcomes will largely depend on personal, cultural, religious, and societal beliefs.

Our study has several strengths. Aside from the long-term follow-up and the large sample size, the data collection was comprehensive and included preadmission status, comorbidities, investigations, and details of organ support. In addition, studies such as this which attempt to elucidate the independent predictors of long-term mortality often run the risk of capturing factors which instead are predictors of in-hospital mortality, simply because of a failure to separate the in-hospital deaths from the deaths after hospital discharge. To circumvent this problem, we used the competing risks which provide a more precise estimation of the event rates and effect measures. The use of standard survival methods such as the Kaplan-Meier analysis has been shown to overestimate cause-specific probabilities in the presence of competing risks [27]. Similarly, in the presence of strong competing risks especially among the frail or elderly populations, the Cox regression model may substantially overestimate the absolute risk of the event. We also calculated mortality in the elderly patients following discharges compared to the general population in the same age group using SMR, an estimation that is uncommon in most such studies due to lack of standardized population.

Our study also has several weaknesses. First, being a single-center study involving a medical ICU, our findings may not be generalizable to other ICU populations. Nonetheless, medical ICUs admit nonelective patients who may therefore be more representative of the elderly population who do not undergo elective surgical procedures frequently. Second, all studies of elderly patients in the ICU, including ours, have analyzed a cohort of patients already admitted to the ICU, thus preselecting patients who have passed through the stringent ICU admission screening. Because patients with severe comorbidities are underrepresented in these studies, the effect of increasing age on the outcomes may be reduced. Third, our database did not allow us to track our patients' functional and cognitive status and their quality of life before or after discharge from the ICU. Indeed, one previous study has shown that activities of daily living and cognitive impairment are risk factors for mortality in elderly individuals [12]. Fourth, some patients might have received “do not resuscitate or intubate” orders following discharge from the ICU, and these were not documented in the database. Fifth, while we used the common threshold of 65 years to define elderly versus nonelderly, we recognize that such a one-dimensional concept of age does not take into account the considerable variability in organ functions and reserves within a certain age group and that others have further subdivided the elderly group, such as 65–75 years for the young-old, 75–80 to 85–90 years for the old-old, and more than 85–90 years for the oldest old [2].

## 5. Conclusion

Elderly ICU patients had a higher in-hospital mortality rate than nonelderly patients, and this difference continued to widen over time after hospital discharge. Predictors of in-hospital mortality included variables associated with the severity of illness such as the need for MV and vasopressors and a higher MPM II score. Predictors for in-hospital mortality which were restricted to the elderly group included malignancy and RRT before admission. In the long-term, factors associated with mortality were a low hemoglobin level on admission, airway disease, and malignancy in both age groups and comorbidities, more so in the nonelderly group. Following hospital discharges elderly patients admitted to ICU have 2.3 times higher mortality compared to the general population of the same age group.

## Figures and Tables

**Table 1:** Baseline characteristics.

Characteristics	Total (*N* = 1563)	Age (year)		*P* value
		<65 (*N* = 854)	≥65 (*N* = 709)	
Gender				<0.001
Male	957 (61.2)	567 (66.4)	390 (55.0)	
Female	606 (38.8)	287 (33.6)	319 (45.0)	
Race				<0.001
Chinese	913 (58.4)	424 (49.7)	489 (69.0)	
Malay	376 (24.1)	228 (26.7)	148 (20.9)	
Indian	163 (10.4)	118 (13.8)	45 (6.4)	
Others	111 (7.1)	84 (9.8)	27 (3.8)	
Comorbidities				<0.001
0-1	468 (30.2)	395 (46.4)	73 (10.5)	
2-3	504 (32.5)	284 (33.4)	220 (31.5)	
>3	577 (37.3)	172 (20.2)	405 (58.0)	
Diagnosis				
Sepsis				
Pulmonary	391 (25.1)	214 (25.1)	177 (25.1)	0.983
Others	103 (6.6)	68 (8.0)	35 (5.0)	0.017
Organ-specific disease				
Hepatic	108 (6.9)	57 (6.7)	51 (7.2)	0.680
Neurological	105 (6.7)	67 (7.2)	44 (6.2)	0.467
Renal	80 (5.1)	38 (4.5)	42 (6.0)	0.185
Cardiovascular	74 (4.8)	31 (3.6)	43 (6.1)	0.023
Endocrine	46 (3.0)	37 (4.3)	9 (1.3)	<0.001
Pulmonary, airway	131 (8.4)	45 (5.3)	86 (12.2)	<0.001
Pulmonary, others^‡^	36 (2.3)	19 (2.2)	17 (2.4)	0.816
Malignancy				0.006
Hematological	68 (4.4)	49 (5.8)	19 (2.7)	
Nonhematological	61 (3.9)	28 (3.3)	33 (4.7)	
Others	193 (12.4)	135 (15.9)	58 (8.2)	<0.001
Treatment				
Mechanical ventilation	986 (63.1)	522 (61.1)	464 (65.4)	0.078
Noninvasive ventilation	620 (39.7)	273 (32.0)	347 (49.0)	<0.001
Vasopressor	745 (47.7)	414 (48.5)	331 (46.7)	0.480
Packed cell transfusion	437 (28.0)	253 (29.6)	184 (26.0)	0.107
Renal replacement therapy				0.003
Preexisting	149 (9.6)	79 (9.3)	70 (9.9)	
New cases	157 (10.1)	106 (12.5)	51 (7.2)	
CPR prior to admission	81 (5.2)	39 (4.6)	42 (5.9)	0.246

CPR: cardiopulmonary resuscitation.

^‡^Specifically obstructive sleep apnea and restrictive lung diseases.

**Table 2:** Characteristics at admissions.

Characteristics	Total (*N* = 1855)	Age (year)		*P* value^*^
		<65 (*N* = 999)	≥65 (*N* = 856)	
Source of admission (%)				
Emergency	1059 (57.1)	576 (57.7)	483 (56.4)	0.301
Ward	751 (40.5)	394 (39.4)	357 (41.7)	
Other	45 (2.43)	29 (2.9)	16 (1.9)	
Investigations				
Hemoglobin (g/dL)	11.6 (9.4–13.5)	11.8 (9.5–13.8)	11.3 (9.4–13.2)	0.028
Platelets/mm^3^	249 (159–345)	246 (143–345)	252 (174.5–344.5)	0.455
White blood cells/mm^3^	12.1 (8.1–17.1)	11.9 (7.8–17.3)	12.3 (8.4–17.1)	0.402
BUN (mmol/L)	8.1 (4.9–14.9)	6.8 (4.3–13.5)	9.8 (5.9–15.7)	<0.001
Creatinine (*µ*mol/L)	104 (68–211)	95 (65–206)	120 (74–217.5)	<0.001
MPM II score	28 (14–45)	19 (9–34)	35 (24–54)	<0.001
LOS (ICU)	3 (2–6)	3 (2–6)	3 (2–5)	1.000
LOS (hospital)	12 (6–23)	12 (6–23)	12 (6–23)	1.000
Reintubation (%)	93 (8.0)	41 (7.6)	52 (8.4)	0.658
Duration from extubation to discharge	33 (25–76)	31 (25–74)	34 (26–78)	0.372
Preexisting kidney disease (%)	397 (21.5)	152 (15.3)	245 (28.6)	<0.001

BUN: blood urea nitrogen; MPM: mortality prediction model; LOS: length of stay; ^*^data provided as number (percentage) and median (interquartile range); significance tests were carried out, accounting for multiple admissions per subject.

**Table 3:** Univariate analysis of mortality in-hospital and following discharge.

Characteristics	Hospital mortality		Mortality following hospital discharge	
	Unadjusted SHR (95% CI)	*P* value	Unadjusted SHR (95% CI)	*P* value
Female sex	0.93 (0.76–1.13)	0.462	1.01 (0.82–1.26)	0.898
Age ≥ 65 years	1.81 (1.49–2.19)	<0.001	1.90 (1.53–2.35)	<0.001
Race		0.163		0.001
Chinese	1.00		1.00	
Malay	0.90 (0.71–1.14)	0.375	1.05 (0.82–1.35)	0.685
Indian	0.67 (0.46–0.96)	0.030	0.71 (0.48–1.04)	0.080
Others	0.88 (0.60–1.30)	0.503	0.21 (0.09–0.48)	<0.001
Comorbidities		0.120		<0.001
0-1	1.00		1.00	
2-3	1.11 (0.86–1.42)	0.428	2.40 (1.69–3.42)	<0.001
>3	1.28 (1.01–1.62)	0.044	3.94 (2.83–5.48)	<0.001
Diagnosis				
Sepsis				
Pulmonary	1.11 (0.90–1.38)	0.335	0.76 (0.59–0.98)	0.037
Others	1.20 (0.84–1.71)	0.315	0.46 (0.25–0.84)	0.011
Organ-specific disease				
Hepatic	0.94 (0.65–1.36)	0.743	1.52 (1.05–2.20)	0.025
Neurological	1.51 (1.06–2.13)	0.021	0.77 (0.48–1.25)	0.289
Renal	0.56 (0.32–0.96)	0.036	2.32 (1.63–3.32)	<0.001
Cardiovascular	0.93 (0.57–1.51)	0.768	1.10 (0.67–1.78)	0.713
Endocrine	0.21 (0.07–0.64)	0.006	0.57 (0.26–1.28)	0.176
Pulmonary, airway	0.55 (0.35–0.86)	0.008	1.74 (1.27–2.40)	0.001
Pulmonary, others^*^	0.69 (0.33–1.43)	0.315	1.67 (0.96–2.93)	0.072
Malignancy		<0.001		0.022
Hematological	2.18 (1.60–2.98)	<0.001	0.97 (0.57–1.65)	0.916
Nonhematological	2.43 (1.68–3.53)	<0.001	1.99 (1.22–3.24)	0.006
Others	0.69 (0.49–0.97)	0.033	0.61 (0.41–0.89)	0.011
Investigations				
Hemoglobin, per g/dL	0.94 (0.91–0.97)	<0.001	0.93 (0.90–0.96)	<0.001
Platelets, per/mm^3^	0.999 (0.998–0.9997)	0.011	1.001 (1.000–1.002)	0.007
White blood cells, per/mm^3^	1.003 (0.999–1.007)	0.104	1.001 (0.996–1.006)	0.773
BUN, per mmol/L	1.006 (0.999–1.014)	0.103	1.010 (1.002–1.018)	0.016
Creatinine, per *µ*mol/L	0.9999 (0.9997–1.0003)	0.922	1.0003 (0.9999–1.0007)	0.062
MPM II score, per point	1.03 (1.02-1.03)	<0.001	1.005 (1.001–1.010)	0.009
Treatment				
Mechanical ventilation	4.66 (3.51–6.19)	<0.001	0.71 (0.57–0.88)	0.002
Noninvasive ventilation	0.75 (0.61–0.91)	0.004	1.62 (1.31–2.01)	<0.001
Vasopressor	4.04 (3.23–5.04)	<0.001	0.58 (0.46–0.72)	<0.001
Packed cell transfusion	1.91 (1.57–2.31)	<0.001	0.98 (0.77–1.24)	0.848
Renal replacement therapy		<0.001		<0.001
Preexisting	2.78 (2.20–3.53)	<0.001	0.54 (0.34–0.86)	0.010
New cases	1.29 (0.95–1.77)	0.107	1.58 (1.16–2.15)	0.004
CPR prior to admission	4.00 (2.94–5.44)	<0.001	0.98 (0.60–1.60)	0.926

SHR: subdistribution hazard ratio; BUN: blood urea nitrogen; CI: confidence interval; CPR: cardiopulmonary resuscitation; MPM: mortality prediction model; ^*^Refers specifically to obstructive sleep apnea and restrictive lung diseases.

**Table 4:** Independent predictors of in-hospital mortality on multivariate analysis.

Characteristics^*^	Adjusted SHR (95% CI)		Adjusted SHR (95% CI)	
	Age < 65 years	*P* value	Age ≥ 65 years	*P* value
Diagnosis				
Neurological disease	1.77 (1.20–2.60)	0.004	1.77 (1.20–2.60)	0.004
Endocrine disease	0.26 (0.08–0.84)	0.024	0.26 (0.08–0.84)	0.024
Malignancy, hematological	0.87 (0.36–2.13)	0.765	3.65 (2.63–5.08)	<0.001
Malignancy, nonhematological	1.17 (0.62–2.18)	0.630	3.40 (2.02–5.73)	<0.001
MPM II score, per point	1.01 (1.01-1.02)	<0.001	1.01 (1.01-1.02)	<0.001
Treatment				
Mechanical ventilation	2.74 (2.00–3.76)	<0.001	2.74 (2.00–3.76)	<0.001
Noninvasive ventilation	0.66 (0.50–0.88)	0.005	0.40 (0.28–0.56)	<0.001
Vasopressor	2.56 (2.00–3.26)	<0.001	2.56 (2.00–3.26)	<0.001
RRT before ICU admission	1.22 (0.83–1.78)	0.317	2.21 (1.62–3.04)	<0.001

SHR: subdistribution hazard ratio; CI: confidence interval; MPM: mortality prediction model; RRT: renal replacement therapy; ^*^only variables which were independently associated with in-hospital mortality (*P* < 0.05) on multivariate analysis are shown.

**Table 5:** Independent predictors of mortality following hospital discharge on multivariate analysis.

	Adjusted SHR (95% CI)			
	Age < 65	*P* value	Age ≥ 65	*P* value
Comorbidities				
2 to 3	2.34 (1.48–3.69)	<0.0001	1.43 (0.75–2.71)	0.275
>3	5.01 (3.21–7.83)	<0.0001	1.86 (1.02–3.40)	0.043
Hb	0.94 (0.90–0.98)	0.002	0.94 (0.90–0.98)	0.002
Diagnosis				
Airway	2.23 (1.57–3.15)	<0.0001	2.23 (1.57–3.15)	<0.0001
Malignancy				
Hematological disease	1.11 (0.63–1.94)	0.721	1.11 (0.63–1.94)	0.721
Nonhematological disease	2.31 (1.39–3.84)	0.001	2.31 (1.39–3.84)	0.001

CI: confidence interval; SHR: subdistribution hazard ratio; Hb: hemoglobin; ^*^only variables which were independently associated with mortality following hospital discharge (*P* < 0.05) on multivariate analysis are shown.

**Table 6:** Standardized mortality ratio of the two groups after hospital discharge.

Mortality following hospital discharge				
Age group	Number of patients in study cohort	Age-adjusted mortality per 1000 in standard population	Number of deaths	Total number of deaths expected
15–19	20	0.2	1	0.004
20–24	64	0.3	5	0.0192
25–29	56	0.3	0	0.0168
30–34	53	0.4	2	0.0212
35–39	47	0.5	6	0.0235
40–44	66	0.9	7	0.0594
45–49	101	1.7	21	0.1717
50–54	163	2.8	31	0.4564
55–59	134	4.4	30	0.5896
60–64	150	7	35	1.05
Age group < 65	**854**		**138**	**2.4118**

Expected mortality over 3.1 years = 2.41 × 3.1 = 7.47				
SMR = 138/7.47 = 18.47				

65–69	162	12.6	42	2.0412
70–74	175	19.9	44	3.4825
75–79	186	37	59	6.882
80–84	105	57.4	33	6.027
85+	81	116.4	21	9.4284
Age group ≥ 65	**709**	**199**	**27.8611**

Expected mortality over 3.1 years = 27.86 × 3.1 = 86.37				
SMR = 199/86.37 = 2.3				

SMR: standardized mortality ratio.

## Abbreviations

ICU: Intensive care unit
HDU: High dependency unit
ICIP: IntelliVue Information Portfolio
MV: Mechanical ventilation
NIV: Noninvasive ventilation
RRT: Renal replacement treatment
MPM: Mortality prediction model
CPSS: Computerized patient support system
SHR: Subdistribution hazard ratio
CI: Confidence interval
SMR: Standardized mortality ratio
IQR: Interquartile range
APACHE: Acute physiology and chronic health evaluation
COPD: Chronic obstructive pulmonary disease.
